# Perceived Impact of COVID on Smoking, Vaping, Alcohol and Cannabis Use Among Youth and Youth Adults in Canada

**DOI:** 10.1177/07067437211042132

**Published:** 2021-09-06

**Authors:** Michael Chaiton, Jolene Dubray, Anasua Kundu, Robert Schwartz

**Affiliations:** 1 Dalla Lana School of Public Health, 7938 University of Toronto, Toronto, Ontario; 2 Centre for Addiction and Mental Health, Toronto, Ontario

**Keywords:** COVID, adolescence, addictions

## Introduction

The recent Coronavirus Disease 2019 (COVID-19) pandemic has affected the mental health of Canadians adversely^1,2^ and as a result, both mental health problems and substance use among adolescents have increased.^2,3^ A recent study reported that 22.6% of Canadian adults increased their alcohol consumption, including 4.8% heavy drinking, compared to their pre-pandemic alcohol consumption levels.^4^ Moreover, the frequency of both alcohol and cannabis use among adolescents has increased as well.^3^ In the United States, retail sales of alcohol and tobacco increased by 34% and 13% respectively during the early pandemic period compared to the pre-pandemic time.^5^ In addition, about half of adult cannabis users in Canada indicated that they have increased their cannabis consumption during the pandemic.^6^

There is limited evidence of changes in substance use among adolescents^2,3^ and adults^4,6^ in response to the COVID-19 pandemic in Canada. Exploring patterns of changes in substance use among youth and young adults is crucial and may have implications for future burden of disease.

## Materials and Methods

### Study Population

Youth and young adults aged 16–25 living in Canada were eligible for the survey. For this cohort, we recruited through Instagram and Facebook advertisements from August 2020 to March 2021 using a variety of recruitment messages. Almost half of each province's sample was recruited in October and November 2020. Overall, 7,745 participants completed the survey. Post-stratification weights were developed from Census data, resulting in the exclusion of 94 participants with incomplete demographic data and those who resided in Yukon, Northwest Territories and Nunavut (total *n* = 7,651). The analytic sample was restricted to 6,721 (87%) participants who reported using at least one of cigarettes, e-cigarettes, alcohol or cannabis during past year.

### Measures

Each participant was asked “How has the COVID-19 Pandemic influenced your use of each of the following …? Increased, Decreased, Unchanged, Did not Use” for cigarettes, e-cigarettes, cannabis, and alcohol. Participants were also asked about their current use of cigarettes, e-cigarettes, cannabis, and alcohol. Demographics (age in years, sex at birth, highest level of education completed, marital status, parental status, and race) were self-reported.

### Analysis

Percentages of participants who reported increases in their use of substances were calculated among participants who reported use of at least one substance. Bivariate associations between demographic characteristics of participants who reported increases in use compared to those who reported decreases/no change /did not use that substance were tested. Logistic regression was used to identify independent demographic predictors of increases in use in any substance. All analysis were conducted in Stata 14 using survey procedures and poststratification weights.

## Results

The unweighted sample was 21% male, 69% under 20 years old, and 74% White (*n* = 7,651). The socio-demographic characteristics of the study population are presented in the Supplementary material 1. Of the 6,721 participants who reported currently using substances in their lifetime, 59% reported increasing their use of at least one substance, 55% reported increasing use of two or more substances. Comparatively, only 16% of lifetime users reported reducing their use of any substance.

Current daily users of substances were most likely to report increased use. Daily cigarette smokers (81%), e-cigarettes vapers (75%), cannabis users (73%) and alcohol users (72%) reported an increase in use. Reported net increases in use overall (percent of users increasing use subtracting percent of users decreasing) during the COVID-19 pandemic were observed for cigarettes (+16%), e-cigarettes (+37%), alcohol (+19%) and cannabis (+47%).

Females, those with a university degree, and people married or living with a partner were independent predictors of increased substance use (Table 1). Youth living in British Columbia and Prince Edward Island were more likely to report increased use compared to youth in Ontario. Indigenous youth were most likely to report increased use, as well as youth from South Asian and Latin American backgrounds, compared to nonvisible minority youth. Youth from Chinese and Southeast Asian backgrounds were less likely to report increased substance use.

**Table 1.** Predictors of Increase in Substance Use Due to the Coronavirus Disease 2019 (COVID-19) Pandemic Among Youth and Young Adults Using at Least One Substance (*n* = 6,721).

Characteristic	OR (95% CI)
Age	1.02
	(0.93,1.12)
Sex (ref: male)	0.48***
	(0.36,0.64)
Education (ref: less than high school)	0.60***
	(0.49,0.73)
Race (ref: White)	
Black	0.75
	(0.35,1.61)
Chinese	0.17***
	(0.07,0.38)
Filipino	0.45*
	(0.22,0.92)
Indigenous	3.16**
	(1.39,7.21)
Japanese	0.34*
	(0.12,0.99)
Korean	1.55
	(0.75,3.21)
Latin-Central-South American	2.90**
	(1.49,5.64)
Southeast Asian	0.06***
	(0.02,0.17)
South Asian	3.10**
	(1.56,6.18)
West Asian	0.88
	(0.45,1.73)
Another Background	1.5
	(0.72,3.15)
Married (ref: Single)	5.00***
	(3.54,7.06)
Parent/legal guardian of children (ref. no children)	1.37
	(0.57,3.31)
Province (ref. Ontario)	
Alberta	1.01
	(0.54,1.90)
British Columbia	3.34**
	(1.34,8.34)
Manitoba	0.71
	(0.38,1.31)
New Brunswick	0.03***
	(0.01,0.05)
Newfoundland and labrador	1.82
	(0.98,3.39)
Nova Scotia	1.06
	(0.50,2.24)
Prince Edward Island	2.04*
	(1.11,3.77)
Quebec	0.59
	(0.30,1.17)
Askatchewan	0.63
	(0.31,1.26)
Date of survey	1
	(1.00,1.00)

*Note:* OR: exponentiated coefficients; CI: confidence intervals.

*P < 0.05, **P < 0.01, ***P < 0.001.

## Discussion

In this survey, most youth and young adults aged 16–25 years old in Canada who used substances reported an increase in the use of one of more substances during the COVID-19 pandemic. The fact that Ontario had a stricter lockdown measures than British Columbia and Prince Edward Island during October and November 2020, when half of the study participants were recruited, may contribute to the higher likelihood of substance use among youth residing in the latter provinces. Use of any substance long-term is associated with health risks, and the dramatic increase in use may lead to tremendous health concerns without immediate action. This study did not have representative sampling frame; however, post-stratification weights were used to match the sample to the characteristics of the Canadian population. Additionally, we did not evaluate the magnitude of the increases or decreases in youth. Further research is needed to examine longitudinal changes in substance use among youth and young adults over time.

## Supplemental Material

sj-docx-1-cpa-10.1177_07067437211042132 - Supplemental material for Perceived Impact of COVID on Smoking, Vaping, Alcohol and Cannabis Use Among Youth and Youth Adults in Canada Click here for additional data file. Supplemental material, sj-docx-1-cpa-10.1177_07067437211042132 for Perceived Impact of COVID on Smoking, Vaping, Alcohol and Cannabis Use Among Youth and Youth Adults in Canada by Michael Chaiton, Jolene Dubray, Anasua Kundu and Robert Schwartz in The Canadian Journal of Psychiatry
