# Supplementary material for: Perceived Impact of COVID on Smoking, Vaping, Alcohol and Cannabis Use Among Youth and Youth Adults in Canada
Source: Can J Psychiatry. 2021 Sep 6;67(5):407–9. doi: 10.1177/07067437211042132 (PMC9065487; doi:10.1177/07067437211042132)
Supplement: sj-docx-1-cpa-10.1177_07067437211042132 - Supplemental material for Perceived Impact of COVID on Smoking, Vaping, Alcohol and Cannabis Use Among Youth and Youth Adults in Canada [file sj-docx-1-cpa-10.1177_07067437211042132.docx]

Supplementary material 1. Socio-demographic characteristics of the participants (n=6721).

| Characteristics | Ontario | Alberta | British Columbia | Manitoba | New Brunswick | Newfoundland and Labrador | Nova Scotia | Prince Edward Island | Quebec | Saskatchewan | Total |
| --- | --- | --- | --- | --- | --- | --- | --- | --- | --- | --- | --- |
|  | n(%) | n(%) | n(%) | n(%) | n(%) | n(%) | n(%) | n(%) | n(%) | n(%) | n(%) |
| **Age (years)** | | | | | | | | | | | |
| 16 | 182  (5.7) | 67  (6.4) | 95  (9.2) | 25  (9.5) | 6  (4) | 5  (4.4) | 22  (8.4) | 6  (11.5) | 34  (9.1) | 19  (8.1) | 461 (6.9) |
| 17 | 356  (11.2) | 150  (14.3) | 146  (14.2) | 25  (9.5) | 12  (7.9) | 16  (14.2) | 22  (8.4) | 6  (11.5) | 63  (16.8) | 26  (11) | 822 (12.2) |
| 18 | 273  (8.6) | 141  (13.5) | 79  (7.7) | 21  (8) | 10  (6.6) | 8  (7.1) | 21  (8) | 6  (11.5) | 49  (13.1) | 28  (11.9) | 636 (9.5) |
| 19 | 452  (14.2) | 135  (12.9) | 141  (13.7) | 33  (12.5) | 22  (14.6) | 14  (12.4) | 32  (12.2) | 5  (9.6) | 36  (9.6) | 32  (13.6) | 902 (13.4) |
| 20 | 346  (10.8) | 113  (10.8) | 114  (11.1) | 24  (9.1) | 24  (15.9) | 16  (14.2) | 28  (10.6) | 4  (7.7) | 31  (8.3) | 26  (11) | 726 (10.8) |
| 21 | 344  (10.8) | 104  (9.9) | 103  (10) | 28  (10.6) | 14  (9.3) | 11  (9.7) | 25  (9.5) | 6  (11.5) | 45  (12) | 28  (11.9) | 708 (10.5) |
| 22 | 342  (10.7) | 96  (9.2) | 98  (9.5) | 28  (10.6) | 20  (13.2) | 9  (8) | 30  (11.4) | 4  (7.7) | 31  (8.3) | 23  (9.7) | 681 (10.1) |
| 23 | 338  (10.6) | 79  (7.5) | 93  (9) | 28  (10.6) | 16  (10.6) | 13  (11.5) | 30  (11.4) | 5  (9.6) | 34  (9.1) | 28  (11.9) | 664 (9.9) |
| 24 | 302  (9.5) | 98  (9.4) | 89  (8.7) | 28  (10.6) | 16  (10.6) | 10  (8.8) | 35  (13.3) | 2  (3.8) | 28  (7.5) | 13  (5.5) | 621 (9.2) |
| 25 | 257  (8.1) | 64  (6.1) | 70  (6.8) | 24  (9.1) | 11  (7.3) | 11  (9.7) | 18  (6.8) | 8  (15.4) | 24  (6.4) | 13  (5.5) | 500 (7.4) |
| **Sex** | | | | | | | | | | | |
| Male | 638  (20) | 244  (23.3) | 244  (23.7) | 53  (20.1) | 30  (19.9) | 26  (23) | 54  (20.5) | 12  (23.1) | 112  (29.9) | 49  (20.8) | 1462  (21.8) |
| Female | 2554  (80) | 803  (76.7) | 784  (76.3) | 211  (79.9) | 121  (80.1) | 87  (77) | 209  (79.5) | 40  (76.9) | 263  (70.1) | 187  (79.2) | 5259  (78.2) |
| **Highest Education level** | | | | | | | | | | | |
| Some elementary or high school | 513  (16.1) | 195  (18.6) | 222  (21.6) | 50  (18.9) | 19  (12.6) | 20  (17.7) | 50  (19) | 13  (25) | 44  (11.7) | 50  (21.2) | 1176 (17.5) |
| Completed High school | 1491  (46.7) | 554  (52.9) | 492  (47.9) | 139  (52.7) | 78  (51.7) | 50  (44.2) | 110  (41.8) | 21  (40.4) | 196  (52.3) | 116  (49.2) | 3247 (48.3) |
| Some college or University | 335  (10.5) | 102  (9.7) | 111  (10.8) | 27  (10.2) | 24  (15.9) | 17  (15) | 36  (13.7) | 6  (11.5) | 77  (20.5) | 24  (10.2) | 759 (11.3) |
| University or post graduate degree | 853  (26.7) | 196  (18.7) | 203  (19.7) | 48  (18.2) | 30  (19.9) | 26  (23) | 67  (25.5) | 12  (23.1) | 58  (15.5) | 46  (19.5) | 1539 (22.9) |
| **Race** | | | | | | | | | | | |
| White | 2393  (75) | 843  (80.5) | 785  (76.4) | 204  (77.3) | 143  (94.7) | 103  (91.2) | 234  (89) | 44  (84.6) | 303  (80.8) | 195  (82.6) | 5247  (78.1) |
| Black | 80  (2.5) | 12  (1.1) | 7  (0.7) | 3  (1.1) | 0  (0) | 1  (0.9) | 3  (1.1) | 0  (0) | 5  (1.3) | 1  (0.4) | 112  (1.7) |
| Chinese | 178  (5.6) | 40  (3.8) | 79  (7.7) | 4  (1.5) | 1  (0.7) | 0  (0) | 2  (0.8) | 0  (0) | 12  (3.2) | 2  (0.8) | 318  (4.7) |
| Filipino | 45  (1.4) | 25  (2.4) | 12  (1.2) | 8  (3) | 0  (0) | 0  (0) | 2  (0.8) | 0  (0) | 1  (0.3) | 3  (1.3) | 96  (1.4) |
| Indigenous | 55  (1.7) | 39  (3.7) | 35  (3.4) | 26  (9.8) | 1  (0.7) | 6  (5.3) | 6  (2.3) | 1  (1.9) | 7  (1.9) | 18  (7.6) | 194  (2.9) |
| Japanese | 4  (0.1) | 0  (0) | 3  (0.3) | 0  (0) | 0  (0) | 0  (0) | 0  (0) | 0  (0) | 1  (0.3) | 0  (0) | 8  (0.1) |
| Korean | 15  (0.5) | 3  (0.3) | 5  (0.5) | 0  (0) | 0  (0) | 0  (0) | 0  (0) | 1  (1.9) | 1  (0.3) | 1  (0.4) | 26  (0.4) |
| Latin-Central-South American | 50  (1.6) | 15  (1.4) | 22  (2.1) | 4  (1.5) | 0  (0) | 1  (0.9) | 2  (0.8) | 2  (3.8) | 14  (3.7) | 2  (0.8) | 112  (1.7) |
| Southeast Asian | 26  (0.8) | 8  (0.8) | 11  (1.1) | 1  (0.4) | 1  (0.7) | 0  (0) | 2  (0.8) | 1  (1.9) | 5  (1.3) | 0  (0) | 55  (0.8) |
| South Asian | 184  (5.8) | 26  (2.5) | 31  (3) | 8  (3) | 1  (0.7) | 1  (0.9) | 2  (0.8) | 1  (1.9) | 2  (0.5) | 4  (1.7) | 260  (3.9) |
| West Asian | 60  (1.9) | 5  (0.5) | 11  (1.1) | 2  (0.8) | 1  (0.7) | 0  (0) | 1  (0.4) | 1  (1.9) | 12  (3.2) | 2  (0.8) | 95  (1.4) |
| Another Background | 102  (3.2) | 31  (3) | 27  (2.6) | 4  (1.5) | 3  (2) | 1  (0.9) | 9  (3.4) | 1  (1.9) | 12  (3.2) | 8  (3.4) | 198  (2.9) |
| **Marital status** | | | | | | | | | | | |
| Single | 2583  (80.9) | 804  (76.8) | 795  (77.3) | 202  (76.5) | 112  (74.2) | 84  (74.3) | 177  (67.3) | 40  (76.9) | 294  (78.4) | 181  (76.7) | 5272  (78.4) |
| Married or living with a partner | 604  (18.9) | 240  (22.9) | 230  (22.4) | 62  (23.5) | 39  (25.8) | 29  (25.7) | 86  (32.7) | 12  (23.1) | 79  (21.1) | 55  (23.3) | 1436  (21.4) |
| Divorced/Separated/Widowed | 5  (0.2) | 3  (0.3) | 3  (0.3) | 0  (0) | 0  (0) | 0  (0) | 0  (0) | 0  (0) | 2  (0.5) | 0  (0) | 13  (0.2) |
| **Parent/ legal guardian of children** | | | | | | | | | | | |
| Yes | 93  (2.9) | 34  (3.2) | 26  (2.5) | 16  (6.1) | 15  (9.9) | 6  (5.3) | 17  (6.5) | 1  (1.9) | 6  (1.6) | 14  (5.9) | 228  (3.4) |
| No | 3099  (97.1) | 1013  (96.8) | 1002  (97.5) | 248  (93.9) | 136  (90.1) | 107  (94.7) | 246  (93.5) | 51  (98.1) | 369  (98.4) | 222  (94.1) | 6493  (96.6) |
| **Cigarette use** | | | | | | | | | | | |
| Daily or almost daily | 290  (9.1) | 90  (8.6) | 86  (8.4) | 24  (9.1) | 17  (11.3) | 10  (8.8) | 36  (13.7) | 7  (13.5) | 35  (9.3) | 26  (11) | 621  (9.2) |
| At least once in the past month | 355  (11.1) | 115  (11) | 139  (13.5) | 38  (14.4) | 20  (13.2) | 19  (16.8) | 31  (11.8) | 7  (13.5) | 46  (12.3) | 27  (11.4) | 797  (11.9) |
| At least once in my life | 866  (27.1) | 344  (32.9) | 291  (28.3) | 72  (27.3) | 49  (32.5) | 39  (34.5) | 75  (28.5) | 18  (34.6) | 107  (28.5) | 81  (34.3) | 1942  (28.9) |
| Never | 1681  (52.7) | 498  (47.6) | 512  (49.8) | 130  (49.2) | 65  (43) | 45  (39.8) | 121  (46) | 20  (38.5) | 187  (49.9) | 102  (43.2) | 3361  (50) |
| **Cannabis use** | | | | | | | | | | | |
| Daily or almost daily | 911  (28.5) | 333  (31.8) | 304  (29.6) | 76  (28.8) | 60  (39.7) | 43  (38.1) | 74  (28.1) | 13  (25) | 94  (25.1) | 69  (29.2) | 1977  (29.4) |
| At least once in the past month | 766  (24) | 248  (23.7) | 254  (24.7) | 58  (22) | 31  (20.5) | 23  (20.4) | 71  (27) | 20  (38.5) | 107  (28.5) | 47  (19.9) | 1625  (24.2) |
| At least once in my life | 756  (23.7) | 242  (23.1) | 265  (25.8) | 62  (23.5) | 31  (20.5) | 27  (23.9) | 62  (23.6) | 5  (9.6) | 91  (24.3) | 62  (26.3) | 1603  (23.9) |
| Never | 759  (23.8) | 224  (21.4) | 205  (19.9) | 68  (25.8) | 29  (19.2) | 20  (17.7) | 56  (21.3) | 14  (26.9) | 83  (22.1) | 58  (24.6) | 1516  (22.6) |
| **E-cigarette use** | | | | | | | | | | | |
| Daily or almost daily | 879  (27.5) | 415  (39.6) | 357  (34.7) | 84  (31.8) | 57  (37.7) | 48  (42.5) | 85  (32.3) | 23  (44.2) | 131  (34.9) | 113  (47.9) | 2192  (32.6) |
| At least once in the past month | 378  (11.8) | 127  (12.1) | 130  (12.6) | 28  (10.6) | 22  (14.6) | 9  (8) | 25  (9.5) | 7  (13.5) | 43  (11.5) | 20  (8.5) | 789  (11.7) |
| At least once in my life | 776  (24.3) | 224  (21.4) | 243  (23.6) | 57  (21.6) | 28  (18.5) | 31  (27.4) | 69  (26.2) | 6  (11.5) | 74  (19.7) | 47  (19.9) | 1555  (23.1) |
| Never | 1159  (36.3) | 281  (26.8) | 298  (29) | 95  (36) | 44  (29.1) | 25  (22.1) | 84  (31.9) | 16  (30.8) | 127  (33.9) | 56  (23.7) | 2185  (32.5) |
| **Alcohol use** | | | | | | | | | | | |
| Daily or almost daily | 306  (9.6) | 88  (8.4) | 98  (9.5) | 13  (4.9) | 9  (6) | 6  (5.3) | 23  (8.7) | 7  (13.5) | 36  (9.6) | 18  (7.6) | 604  (9) |
| At least once in the past month | 2063  (64.6) | 701  (67) | 671  (65.3) | 179  (67.8) | 104  (68.9) | 82  (72.6) | 180  (68.4) | 35  (67.3) | 253  (67.5) | 168  (71.2) | 4436  (66) |
| At least once in my life | 738  (23.1) | 229  (21.9) | 230  (22.4) | 67  (25.4) | 36  (23.8) | 24  (21.2) | 53  (20.2) | 10  (19.2) | 80  (21.3) | 43  (18.2) | 1510  (22.5) |
| Never | 85  (2.7) | 29  (2.8) | 29  (2.8) | 5  (1.9) | 2  (1.3) | 1  (0.9) | 7  (2.7) | 0  (0) | 6  (1.6) | 7  (3) | 171  (2.5) |
